# Regional Inequalities in Trachoma Prevention in Ethiopia: A Meta‐Analysis

**DOI:** 10.1002/hsr2.72416

**Published:** 2026-04-20

**Authors:** Zufan Alamrie Asmare, Almaw Genet Yeshiwas, Gashaw Melkie Bayeh, Tilahun Degu Tsega, Sintayehu Simie Tsega, Asaye Alamneh Gebeyehu, Getaneh Atikilt Yemata, Rahel Mulatie Anteneh, Amare Genetu Ejigu, Ahmed Fentaw Ahmed, Zeamanuel Anteneh Yigzaw, Abathun Temesgen, Abebaw Molla Kebede, Habitamu Mekonen, Anley Shiferaw Enawgaw, Getasew Yirdaw, Berhanu Abebaw Mekonnen, Meron Asmamaw Alemayehu, Zekaryas Ewnetu Gashu, Chalachew Yenew

**Affiliations:** ^1^ Department of Ophthalmology, School of Medicine and Health Science Debre Tabor University Debre Tabor Ethiopia; ^2^ Department of Environmental Health, College of Medicine and Health Science Injibara University Injibara Ethiopia; ^3^ Department of Public Health, College of Medicine and Health Sciences Injibara University Injibara Ethiopia; ^4^ Department of Epidemiology, School of Public Health, Cheeloo College of Medicine Shandong University Jinan China; ^5^ Department of Medical Nursing, School of Nursing, College of Medicine and Health Science University of Gondar Gondar Ethiopia; ^6^ Department of Public Health, College of Health Science Debre Tabor University Debre Tabor Ethiopia; ^7^ Department of Midwifery, College of Medicine and Health Sciences Injibara University Injibara Ethiopia; ^8^ Facility of Health Science and Technology University of Brazilia Brazilia Brazil; ^9^ Department of Health Promotion and Behavioral Sciences, School of Public Health, College of Medicine and Health Sciences Bahir Dar University Bahir Dar Ethiopia; ^10^ Department of Human Nutrition, College of Health Science Debre Markos University Debre Markos Ethiopia; ^11^ Department of Public Health, College of Health Sciences Debre Markos University Debre Markos Ethiopia; ^12^ Department of Environmental Health Science, College of Medicine and Health Sciences Debre Markos University Debre Markos Ethiopia; ^13^ Department of Nutrition and Dietetics, School of Public Health, College of Medicine and Health Sciences Bahir Dar University Bahir Dar Ethiopia; ^14^ Department of Epidemiology and Biostatistics, Institute of Public Health, College of Medicine and Health Sciences University of Gondar Gondar Ethiopia; ^15^ Department of Pediatrics and Child Health, College of Medicine and Health Sciences University of Gondar Gondar Ethiopia; ^16^ Department of Public Health Debre Tabor University, Ethiopia, and Research Degree PhD student at Jockey Club College of Veterinary Medicine and Life Sciences, City University of Hong Kong, Hong Kong SAR China

**Keywords:** Ethiopia, prevention practices, public health, regional inequality, systematic review and meta‐analysis, trachoma

## Abstract

**Background and Aims:**

Trachoma remains a significant public health problem in Ethiopia, particularly affecting children. Mothers play a crucial role in implementing preventive practices that interrupt trachoma transmission. Therefore, this systematic review and meta‐analysis seek to address Regional Inequalities in Trachoma Prevention in Ethiopia.

**Methods:**

A comprehensive systematic search was conducted in PubMed, EMBASE, HINARI, Scopus, Google Scholar, and Web of Science for studies published between January 2010 and November 2023. Data extraction and quality assessment were performed using standardized tools. Statistical analysis was conducted using R version 4.5.1 with **meta**, **metafor**, and **ggplot2** packages. Pooled prevalence was estimated using random‐effects meta‐analysis with generalized linear mixed models (GLMM) and logit transformation. Heterogeneity was assessed using the *I*² statistic.

**Results:**

Four cross‐sectional studies, comprising a total of 2240 mothers from diverse regions of Ethiopia, were included in the final analysis. The pooled prevalence of good trachoma prevention practices among mothers was 49% (95% CI: 41–58%). Substantial heterogeneity was observed across studies (*I*² = 91.5%, *p* < 0.001), with regional prevalence ranging from 36% in Tigray to 60% in the Southern Nations, Nationalities, and Peoples' Region (SNNPR). Subgroup analysis by publication year suggested a higher prevalence in studies published in or after 2022 (55%) compared to earlier studies (44%), though this difference was not statistically significant (*p* = 0.1107). Sensitivity analysis confirmed the robustness of the pooled estimate. Publication bias was assessed and deemed non‐significant (Egger's test *p* = 0.351).

**Conclusion:**

This meta‐analysis reveals significant regional inequalities in trachoma prevention practices among Ethiopian mothers, with a pooled national prevalence of 49%, below the level required to interrupt transmission. The high heterogeneity observed underscores the substantial variation in implementation across regions. Future studies should prioritize identifying context‐specific interventions and key modifiable factors to address these disparities and accelerate progress toward elimination.

## 1 Introduction

Trachoma, caused by the bacterium Chlamydia trachomatis, is the leading infectious cause of blindness worldwide. It is hyperendemic in Ethiopia, which carries half of the global trachoma burden [1, 2]. It is transmitted via eye discharge through hands, flies, and contaminated items, and is prevalent in areas with inadequate personal and community hygiene practices [3, 4]. As per the 2018 report of the World Health Organization (WHO) weekly epidemiologic record, the number of people living in precincts where active trachoma was a public health problem was 157.7 million, 88% of which were in Africa, and 50% (69,802,693) of which were in Ethiopia [5]. This cataclysmic disease can provoke vision loss, stigma, curtailed productivity, and economic loss of US$2.9–5.3 billion annually, and half of the global burden of trachoma is attributed to Ethiopia [6, 7].

Africa remains the continent most affected and with the most in‐depth control attempts. In 2017, in the 22 countries of the WHO's Africa Region in which trachoma is purported to be a public health problem, Ethiopia signed the VISION 2020 Initiative in 2002 and augmented its 20‐year strategic plan to eliminate trachoma as a public health problem [8].

The WHO, in alliance with other national health services and non‐governmental organizations (NGOs) inaugurated the implementation of a program delineated as Surgery for advanced disease, Antibiotics, facial cleanliness, and Environmental improvement (SAFE) to wipe out trachoma as a public health problem (Global Elimination of Trachoma by 2020) [9, 10, 11]. Proper prevention practices account for 58.7% and 37.4% of the abatement of trachoma prevalence at all ages and in children, respectively [12]. Mothers, as the primary caregivers of children, the age group most susceptible to active trachoma infection, play a pivotal role in implementing preventive hygiene practices, such as face washing and maintaining a clean household environment. Therefore, understanding the determinants of their Trachoma Prevention Practices (TPP) is critical for effective disease control. Therefore, improvements in hygiene behavior and environmental conditions are exemplary long‐term strategies for trachoma control among mothers [13]. The SAFE strategy endorsed by the World Health Organization—which stands for Surgery, Antibiotics, Facial cleanliness, and Environmental improvement—is fundamental to control efforts against trachoma. The ‘F' (Facial cleanliness) and ‘E' (Environmental improvement) components, which depend on the practices of both the community and individuals, are essential for long‐term success. Mothers, as the primary caregivers of children who are the most vulnerable to active trachoma infection, play a crucial role in implementing these preventive hygiene practices. Studies conducted in Ethiopia have shown that the prevalence of effective Trachoma Prevention Practices (TPP) varies widely, ranging from 35.6% to 59.6% [14, 15, 16, 17]. Evidence shows that basic knowledge and attitudes about trachoma, health education, time spent, and frequency of accessing water were practiced through the TPP [14, 16, 17].

Studies on TPP (Tobacco‐Related Pregnancy Problems) among mothers in Ethiopia have shown inconsistent prevalence rates that vary by region. This systematic review and meta‐analysis aim to determine the national pooled prevalence of TPP and the associated factors among mothers in Ethiopia. Independent studies conducted in various regions of Ethiopia have investigated the prevalence of TPP among mothers and the factors linked to it. However, these studies have demonstrated significant variations and inconsistencies in their findings. To date, the reasons behind these discrepancies have not been systematically explored. Furthermore, previous studies have focused on specific regions and have not provided a comprehensive analysis of maternal determinants and community‐level interventions on a national scale.

Currently, there is no systematically conducted, nationally representative study in Ethiopia to inform evidence‐based policies and enhance intervention programs. Existing epidemiological research has not adequately addressed this gap, given the wide variations in prevalence estimates. Therefore, this systematic review and meta‐analysis seek to address Regional Inequalities in Trachoma Prevention in Ethiopia.

## 2 Materials and Methods

### 2.1 Design and Search Strategy

This systematic review and meta‐analysis aimed to synthesize evidence on trachoma prevention practices among mothers of children aged 1–9 years in Ethiopia, following the Preferred Reporting Items for Systematic Review and Meta‐Analysis (PRISMA) guidelines [18].

Relevant articles on the topic were systematically searched in PubMed/MEDLINE, EMBASE, HINARI, Scopus, Google Scholar, and Web of Science. Additionally, repositories of Ethiopian universities were explored to identify relevant gray literature. Search terms were developed using Medical Subject Headings (MeSH) such as “trachoma prevention practice,” “mothers,” and “Ethiopia,” and these were adapted for use across different databases. The gray literature search included government reports and organizational repositories but deliberately excluded preprints and conference abstracts to maintain the quality and reliability of the included studies. By virtue of the search combinations, we depicted the references of retrieved articles to designate articles that had not been indexed in electronic databases. Two author groups, Group One (H.M., A.S.E., B.A.M., M.A.A.) and Group Two (G.Y., Z.E.G., C.Y.), independently searched the articles. The probing of articles was executed on November 1‐30/2023, using the following search combinations: “Trachoma Prevention Practice”[Title/Abstract] OR “Trachoma Control Strategies “OR “Prevention Intervention” OR” Trachoma Hygiene Practices “[Title/Abstract]) AND Mothers [Title/Abstract] OR Women [Title/Abstract] AND (Ethiopia [Title/Abstract]). We searched for studies published between January 2010 and November 2023.

### 2.2 Eligible Criteria

Inclusion criteria focused on the prevalence question using PCC: P: Population/Patient/Problem (What is the prevalence of TPP?), C: Concept/Phenomena (among mothers of children aged 1–9 years), C: Context (in Ethiopia). (1) studies conducted in Ethiopia; (2) cross‐sectional studies reporting trachoma prevention practices among mothers of children aged 1–9 years; (3) English‐language publications; (4) studies reporting prevalence.

Exclusion criteria included: (1) citations without abstracts/full‐texts; (2) studies not focusing on the specified population or outcomes.

### 2.3 Study Selection and Quality Appraisal

Duplicate removal and systematic screening were performed using EndNote X7. Subsequently, titles and abstracts were independently screened by two groups of reviewers: Group One (Z.A.A., C.Y., G.A.Y.) and Group Two (Z.A.Y., A.T., A.M.K.). Discrepancies between the two groups were resolved through discussion and consensus by a third group of reviewers (A.G.Y., G.M.B., T.D.T., S.S.T., A.A.G.). After duplicate removal, titles and abstracts were screened for relevance. The full texts of potentially eligible studies were then assessed against the inclusion criteria. Any discrepancies were resolved through consensus. Four studies ultimately met the inclusion criteria.

The methodological quality of the included studies was assessed using the Joanna Briggs Institute (JBI) critical appraisal checklists for analytical cross‐sectional studies [19, 20]. Studies scoring 7 or above out of 8 were considered high quality, while those scoring 5‐6 were deemed moderate quality [18].

### 2.4 Outcome Measures

The mother's level of TPP was the primary outcome reported in this systematic review and meta‐analysis.

### 2.5 Data Extraction and Management

Data were extracted using a standardized JBI extraction form in Microsoft Excel, which included the following information: first author, publication year, study region, sample size, response rate, and prevalence of trachoma prevention practices.

### 2.6 Statistical Analysis

A primary outcome analysis employed a random‐effects meta‐analysis using the generalized linear mixed model (GLMM) with a logit transformation to pool prevalence estimates. Heterogeneity was quantified using the *I*² statistic, with values of 25%, 50%, and 75% representing low, moderate, and high heterogeneity, respectively [21].

Subgroup analysis was conducted by publication period (before vs. after 2022) to explore sources of heterogeneity. Sensitivity analysis was performed using the leave‐one‐out method to assess the robustness of pooled estimates [22, 23]. Publication bias assessment included visual inspection of funnel plots and Egger's regression test, though interpretation was cautious given the limited number of studies (< 10) [23]. All analyses were conducted using R statistical software (version 4.5.1) with the meta, metafor, and ggplot2 packages, ensuring comprehensive and reproducible meta‐analytic methods.

## 3 Results

### 3.1 Searching Results

**Figure 1:**
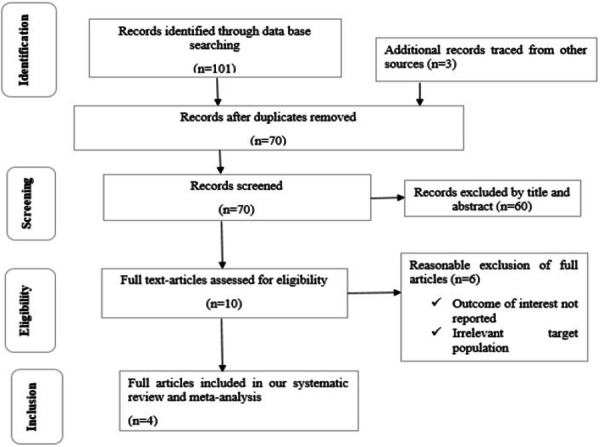
PRISMA 2020 flow diagram for the systematic review of trachoma prevention practices among mothers in Ethiopia.

A total of 104 studies were identified through electronic database searches (*n* = 101) and other sources (*n* = 3). After removing 34 duplicates, 70 records remained. Following title and abstract screening, 60 studies were excluded for not meeting the inclusion criteria. The remaining 10 full‐text articles were assessed for eligibility, and 6 were excluded after full‐text review for not meeting the predefined inclusion criteria. Ultimately, 4 studies met all eligibility requirements and were included in the final systematic review and meta‐analysis (Figure 1).

### 3.2 Description of the Included Studies

As portrayed in Table 1, 4 primary studies were embodied in this systematic review and meta‐analysis. All the included articles reported a total of 2240 mothers, with a response rate ranging from 89.8% [15] to 100% [16]. All were cross‐sectional studies, with a sample size ranging from 209 (15) to 845 [14]. These studies were conducted in various regions of the country, including the Amhara region (17), Tigray (15), Oromia (14), and the Southern Nations, Nationalities, and Peoples' Region (SNNPR) [16]. The lowest and highest magnitude of good trachoma prevention practice among mothers in Ethiopia were 59.6% [16] and 35.6% [15], respectively. Independent appraisers reassessed the articles before the analysis, and they achieved a quality score of 5 or higher. The description of included studies is presented in the subsequent table (Table 1).

**Table 1:** Distribution of studies included in this systematic review and meta‐analysis, 2023.

Study	Region	Year	Study design	Sample size	Response rate	Events (Good TPP)	Proportion	Standard error
Gebretnsae et al. [15]	Tigray	2020	Cross‐sectional	209	89.8	74	0.3560	0.0331
Abera et al. [14]	Oromia	2021	Cross‐sectional	845	94.7	435	0.5150	0.0172
Asmare et al. [17]	Amhara	2023	Cross‐sectional	634	98.42	329	0.4984	0.0199
Shobiso et al. [16]	SNNP	2023	Cross‐sectional	552	100	316	0.5960	0.0209

### 3.3 A Pooled Prevalence of Good Trachoma Prevention Practices

**Figure 2:**
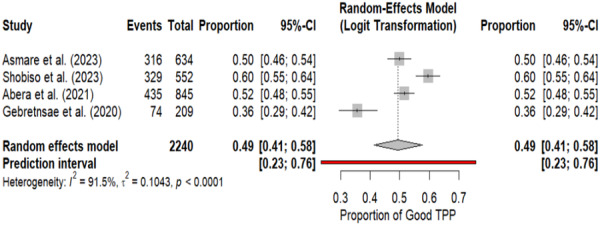
Pooled prevalence and heterogeneity of trachoma prevention practices in Ethiopia: meta‐analysis results.

This meta‐analysis of four studies comprising 2240 participants reveals a pooled prevalence of good trachoma prevention practices of 49% (95% CI: 41–58%) in Ethiopia, though with substantial regional variation ranging from 36% in the Tigray region (Gebreinsae et al., 2020) to 60% in the SNNP region (Shobiso et al., 2023). The extremely high heterogeneity (*I*² = 91.5%, *p* < 0.001) indicates that the observed variation in trachoma prevention practices is unlikely to be due to chance alone but rather reflects genuine differences across regions and study populations. The wide prediction interval (23–76%) further underscores substantial heterogeneity, suggesting that in future settings or populations, the prevalence of good trachoma prevention practices could range from 23% to 76%. These findings highlight the critical importance of contextual factors in trachoma prevention and suggest that while the overall pooled estimate provides a national overview, effective interventions must be tailored to address the specific needs and circumstances of different regions, particularly targeting areas with lower prevention practice rates like Tigray, while learning from higher‐performing regions like SNNP (Figure 2 and Table 1).

### 3.4 Publication Bias Assessment

**Figure 3:**
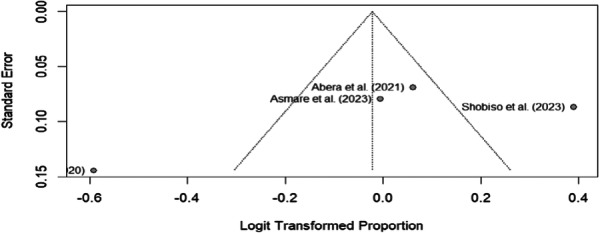
Publication bias assessment.

The funnel plot displays the relationship between study precision (standard error) and effect size (logit‐transformed proportion) for the included studies in the trachoma prevention practices meta‐analysis. The plot displays two studies, Abera et al. (2021) and Shobiso et al. (2023), along with their respective standard errors and logit‐transformed proportions. The two visible studies appear relatively symmetrically distributed around the vertical line representing the overall effect size, though with only two studies, a comprehensive assessment of publication bias is limited. The studies show varying levels of precision (the inverse of the standard error), which is expected in meta‐analyses, where studies with larger sample sizes typically have smaller standard errors. The partial display of only two studies suggests that either Additional studies may be present but not visible in this crop of the funnel plot, or that the funnel plot is incomplete. This may represent a subgroup analysis rather than the complete dataset. With only two clearly visible studies, it is challenging to make definitive conclusions about publication bias. A proper assessment of funnel plots typically requires at least 10 studies to reliably detect asymmetry, which may indicate publication bias. For a more thorough evaluation of publication bias, it is essential to examine the complete funnel plot, including all included studies. Additionally, conducting statistical tests such as Egger's regression test can help objectively assess funnel plot asymmetry. In this case, Egger's test supported the results, with a bias coefficient (B) of −9.4282 (95% CI: 24.23, −43.08) and a *p*‐value of 0.351 (Figure 3).

### 3.5 Subgroup Analysis by Publication Year: Prevalence of Trachoma Prevention Practices

**Figure 4:**
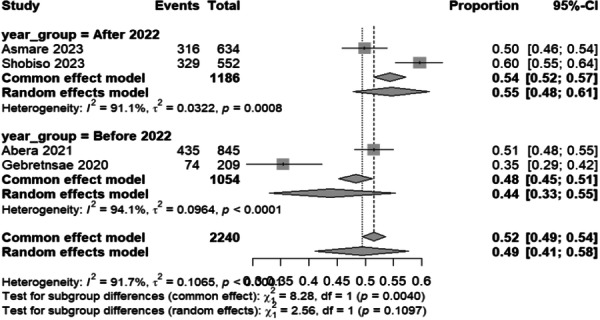
Forest plot of the pooled prevalence of trachoma prevention practice among mothers in Ethiopia by publication year, 2023.

Subgroup analysis by publication year (before vs. after 2022) was conducted to explore temporal trends, coinciding with intensified national trachoma elimination efforts following the COVID‐19 pandemic, research published after 2022 shows a pooled prevalence of 54.7% (95% CI: 47.9–61.4%), which is significantly higher than the 43.8% (95% CI: 33.0–55.3%) found in studies published prior to that year. This represents an absolute increase of 10.9% and a relative increase of 24.9%. However, careful interpretation of these findings is crucial, as the statistical significance varies depending on the model used. The common‐effect model indicates a significant subgroup difference (*p* = 0.004), whereas the random‐effects model yields a non‐significant *p*‐value of 0.11. Both subgroups exhibit high heterogeneity (*I*² > 90%), with the pre‐2022 studies showing a greater between‐study variance (*τ*² = 0.0964 vs. 0.0322). This variability suggests that factors beyond publication significantly influence prevalence estimates (Table 2 and Figure 4). Possible explanations for these trends include increased awareness resulting from enhanced national trachoma elimination programs, methodological differences across studies, and regional variations that may impact sample sizes and statistical power. The increase in TPP prevalence aligns with Ethiopia's intensified trachoma control efforts; however, the lack of statistical significance in the random‐effects model underscores the need for further research to validate this trend. Additionally, the persistent high heterogeneity underscores the need for more nuanced stratification in future analyses, considering variables such as region, intervention type, and measurement methods [11].

**Table 2:** Subgroup analysis of pooled prevalence of trachoma prevention practices by publication year.

Subgroup	Studies (k)	Pooled prevalence (95% CI)	*I*² (Heterogeneity)	*τ*² (tau²)	*p* value (heterogeneity)
After 2022	2	54.7% [47.9–61.4%]	91.1%	0.0322	0.0008
Before 2022	2	43.8% [33.0–55.3%]	94.1%	0.0964	< 0.0001
Overall (All studies)	4	49.4% [41.1–57.7%]	91.7%	0.1065	< 0.0001

*Note:* Test for subgroup differences (random effects model): *χ*² = 2.56, df = 1, *p* = 0.11.

This subgroup analysis examining trachoma prevention practices by study period reveals important temporal patterns, though the differences between subgroups do not reach statistical significance (*p* = 0.1107). Studies conducted in 2022 and later show a higher pooled prevalence of good trachoma prevention practices at 55% (95% CI: 48–61%) than studies before 2022, which show a pooled prevalence of 44% (95% CI: 33–55%). This 11‐percentage‐point difference suggests a potential improvement in trachoma prevention practices over time, possibly reflecting the cumulative impact of ongoing trachoma elimination programs in Ethiopia. However, both subgroups exhibit substantial heterogeneity (*I*² = 91.1% for recent studies and *I*² = 94% for earlier studies), indicating considerable variation within each time period that cannot be explained solely by temporal factors. The high heterogeneity (*I*² = 91.5%) suggests that factors beyond the study period, such as regional differences, implementation variability, and contextual factors, play a significant role in determining trachoma prevention practices. The non‐significant subgroup differences (*p* = 0.1107) indicate that, while there appears to be a trend toward improvement in more recent years, this temporal pattern does not fully account for the substantial variation observed across studies. This reinforces the need for multifaceted interventions that address the complex determinants of trachoma prevention practices beyond simply temporal progression (Figure 4).

### 3.6 Galbraith Plot for Heterogeneity

**Figure 5:**
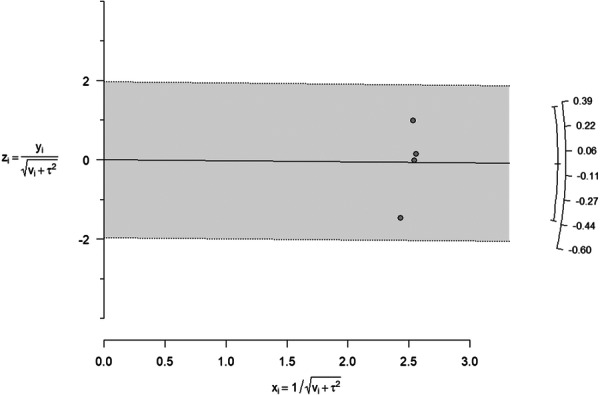
Radial (Galbraith) plot for heterogeneity assessment. Each point represents one included study, plotted by standardized effect size (y‐axis) against precision (x‐axis). The wide scatter around the reference line (dashed) visually confirms high between‐study heterogeneity.

A radial (Galbraith) plot was generated to visually assess heterogeneity, as depicted in Figure 5, revealing a wide scatter of study points around the reference line (representing the pooled logit prevalence) that confirms the substantial statistical heterogeneity quantified by an *I*² statistic of 91.7%. Although all studies fell within acceptable boundaries with no clear outliers identified, it's important to note the limitations of this analysis, including the small number of studies (only four), which may obscure patterns, and the expected high heterogeneity due to regional and methodological diversity. While the radial plot serves as a valuable visual tool, it should complement, not replace, statistical measures, providing insights such as highlighting potential outliers and patterns not evident in forest plots. Clinically, this highlights the need for subgroup analysis and careful interpretation of pooled estimates, ultimately fulfilling a comprehensive assessment of heterogeneity beyond the *I*² statistic alone (Figure 5).

### 3.7 Sensitivity Analysis

The leave‐one‐out sensitivity analysis of pooled trachoma prevention practices demonstrates the robustness of the prevalence estimate, which remains consistent between 48.9% and 53.2% when individual studies are excluded, all within the original 95% confidence interval of 49.5–53.6%. Notably, the study by Shobiso (2023) shows the highest prevalence at 59.6% and its exclusion leads to the lowest pooled estimate, indicating its upward influence, while Gebretnsae (2020), with the lowest prevalence at 35.4%, when omitted, yields a higher pooled estimate of 53.2% and significantly reduces heterogeneity (*I*² = 84.5%), suggesting it is a key contributor to between‐study variability. In contrast, the exclusions by Asmare (2023) and Abera (2021) have minimal impact on the point estimate but increase heterogeneity (*I*² > 94%). Overall, no single study significantly shifts the pooled prevalence by more than ±3.3%, confirming that the estimated prevalence of approximately 50% for good trachoma prevention practices remains statistically significant and within epidemiologically plausible ranges (Table 3).

**Table 3:** Sensitivity analysis using leave‐one‐out method to assess the influence of individual studies on pooled prevalence estimates.

Omitted study	Pooled proportion	95% CI	*τ*²	*Τ*	*I*²	Change from overall
None (All studies)	0.5152	[0.4945; 0.5358]	0.1065	0.3264	91.7%	—
Asmare 2023	0.5218	[0.4973; 0.5462]	0.1505	0.3880	94.3%	+1.28%
Shobiso 2023	0.4887	[0.4649; 0.5126]	0.0675	0.2599	88.4%	−5.14%
Abera 2021	0.5154	[0.4892; 0.5416]	0.1481	0.3849	94.4%	+0.04%
Gebretnsae 2020	0.5318	[0.5100; 0.5534]	0.0222	0.1490	84.5%	+3.22%

*Note:* The “Change from Overall” column displays the percentage change in the pooled proportion compared to the model that includes all studies.

## 4 Discussion

This review found a pooled prevalence of good TPP of 49.5%, which is below the level required to interrupt transmission. This estimate falls within the range reported by the primary studies (35.6–59.6%) but provides a more robust national figure. The finding is consistent with a study from Kenya (50%) but lower than one from Vietnam. This discrepancy may be attributed to differences in access to Water, Sanitation, and Hygiene (WASH) services and the focus of the study population [10]. However, the pooled prevalence of good TPP in this meta‐analysis was lower than in a study conducted in North Vietnam [24]. The plausible explanation might be due to the contraption and inequitable health access, as well as the access to Water, Sanitation, and Hygiene (WASH) services, sewerage, and hygiene vary between countries. Moreover, it might be due to the departures in the study population, as in Vietnam, children under 9 years were the study subjects, and it might be due to the amendments in socio‐demographics, cultures, and study methods [25].

There is an ongoing burden of trachoma in Ethiopia, which remains one of the most endemic countries globally. According to the World Health Organization's 2023 epidemiological update, Ethiopia had 691 districts identified as requiring intervention, with approximately 64 million people living in areas where implementation of the SAFE strategy, Surgery, Antibiotics, Facial cleanliness, and Environmental improvement remains necessary. Despite significant efforts, including the provision of antibiotic treatment to over 18 million people in 2022 and 2023, national antibiotic coverage stood at 27.2%, with only 25.5% of districts achieving the recommended ≥ 80% treatment coverage. These statistics reflect the complexity of eliminating trachoma as a public health problem in high‐burden settings and highlight the need for sustained, multi‐sectoral efforts to address environmental determinants, improve surgical coverage, and enhance community engagement. The subgroup analyses in this report, based on study year and sample size, provide additional insight into heterogeneity and may inform targeted interventions in regions that are lagging behind elimination thresholds [26].

Publication year often reflects changes in national health interventions, while sample size influences the precision and variability of prevalence estimates. Regional differences in TPP may be attributed to disparities in the study period and sample size. In this review, the pooled prevalence of good TPP among mothers was 43.8% in studies conducted before 2022, compared to 54.7% in those conducted after 2022. This apparent increase in recent years may be explained by improvements in public awareness and lifestyle shifts toward more hygienic and health‐conscious practices, potentially influenced by strengthened national trachoma control efforts [27].

### 4.1 Strengths and Limitations of the Study

A key strength of this study is that it provides the first national estimate of TPP among mothers in Ethiopia, synthesizing evidence from multiple regions. The main limitations are the small number of included studies and their cross‐sectional design, which preclude the ability to make causal inferences. Furthermore, restricting the search to English‐language publications may have introduced language bias.

## 5 Conclusion and Recommendations

This systematic review and meta‐analysis reveal that the pooled prevalence of good trachoma prevention practices among mothers of children aged 1–9 years in Ethiopia is 49% (95% CI: 41–58%). This level falls substantially below the threshold required to interrupt trachoma transmission at a national scale and is insufficient to meet the country's goal of eliminating the disease. The analysis uncovered considerable heterogeneity across regions, with prevalence ranging from 36% in Tigray to 60% in SNNPR, underscoring the significant influence of local contextual, socioeconomic, and programmatic factors on the implementation of the facial cleanliness (‘F’) and environmental improvement (‘E’) components of the SAFE strategy.

These findings highlight a critical gap in current prevention efforts. Future research should prioritize investigating the specific determinants that impact these practices to inform effective, targeted interventions. Aligning these efforts with Ethiopia's national strategy and the global SAFE framework is essential to accelerate progress toward eliminating trachoma as a public health problem.

## Author Contributions

**Zufan Alamrie Asmare:** conceptualization, investigation, writing – review and editing, writing – original draft. **Tilahun Degu Tsega:** writing – original draft, writing – review and editing, formal analysis. **Sintayehu Simie Tsega:** conceptualization, writing – original draft, writing – review and editing. **Getaneh Atikilt Yemata:** writing – original draft, writing – review and editing, validation. **Amare Genetu Ejigu:** investigation, writing – original draft, writing – review and editing. **Ahmed Fentaw Ahmed:** investigation, writing – original draft, writing – review and editing. **Zeamanuel Anteneh Yigzaw:** writing – original draft, writing – review and editing, visualization. **Habitamu Mekonen:** conceptualization, writing – original draft, writing – review and editing. **Getasew Yirdaw:** methodology, writing – original draft, writing – review and editing. **Zekaryas Ewnetu Gashu:** conceptualization, methodology, writing – review and editing, writing – original draft. **Chalachew Yenew:** conceptualization, investigation, writing – original draft, methodology, writing – review and editing, formal analysis, data curation, supervision, resources, software.

## Funding

The authors have nothing to report.

## Ethics Statement

This study is a systematic review; therefore, it did not require ethics approval or consent to participate.

## Consent

The authors have nothing to report.

## Conflicts of Interest

The authors declare no conflicts of interest.

## 1 Transparency Statement

The lead author, Chalachew Yenew, affirms that this manuscript is an honest, accurate, and transparent account of the study being reported; that no important aspects of the study have been omitted; and that any discrepancies from the study as planned (and, if relevant, registered) have been explained.

## Data Availability

The data that support the findings of this study are available from the corresponding author upon reasonable request.
